# Prevalence of *BRCA1* and *BRCA2* Variants in an Unselected Population of Women With Breast Cancer

**DOI:** 10.1001/jamanetworkopen.2025.31577

**Published:** 2025-09-15

**Authors:** Joanne E. Mortimer, Sidney S. Lindsey, Elyssa Zukin, Wai Park, Duveen Sturgeon, Ilana Solomon, Kathleen Blazer, Stacy W. Gray, Joseph D. Bonner, Stephen B. Gruber

**Affiliations:** 1 Department of Medical Oncology and Therapeutics Research, City of Hope National Medical Center, Duarte, California; 2 Center for Precision Medicine, City of Hope National Medical Center and Beckman Research Institute, Duarte, California; 3 Cedars Sinai, Los Angeles, California

## Abstract

**Importance:**

Barriers to germline testing make it difficult to determine the prevalence of pathogenic or likely pathogenic (P/LP) variants in *BRCA1* and *BRCA2* in underrepresented populations.

**Objective:**

To determine P/LP variants in *BRCA1* and *BRCA2* in women with breast cancer enrolled in the Implementing Next-Generation Sequencing for Precision Intervention and Risk Evaluation (INSPIRE) study.

**Design, Setting, and Participants:**

This cohort study included patients from City of Hope Duarte, California, and Upland, California, with a breast cancer diagnosis of any stage (0 to IV) from July 2020 to October 2023 who were enrolled in the INSPIRE biorepository study. Blood, buccal smears, or saliva were collected for germline testing of 155 genes. Data were analyzed from October 2023 to December 2024.

**Exposures:**

Women with breast cancer, including underrepresented populations, received germline testing to determine P/LP variants in *BRCA1* and *BRCA2* genes.

**Main Outcomes and Measures:**

The main outcome was prevalence of P/LP variants in *BRCA1* and *BRCA2* in women with breast cancer.

**Results:**

A total of 2401 women with breast cancer underwent germline testing, including 136 African American or Black (5.7%), 365 Asian (15.2%), 15 American Indian or Alaska Native women (0.5%), 8 Native Hawaiian or Other Pacific Islander women (0.3%), and 1666 White (69.4%), 86 Other (3.6%), and 125 unknown (5.2%). Ethnicity was reported for 737 Hispanic or Latina women (30.7%) and 1545 non-Hispanic women (64.3%). P/LP variants in *BRCA1* or *BRCA2* were identified in 16 Asian women (4.4%), 11 African American or Black women (8.1%), and 80 White women (4.8%). Hispanic women with breast cancer were 2.58 times as likely as non-Hispanic women to carry a P/LP germline variant in *BRCA1* than *BRCA2* variants (odds ratio [OR], 2.58 [95% CI, 1.16-5.88]; *P* = .02). P/LP variants in *BRCA1* were more likely to be identified in Hispanic women (22 of 737 [3.0%]) than in non-Hispanic women (25 of 1545 [1.6%]) (OR, 1.87; [95% CI, 1.04-3.34]; *P* = .02). Additionally, 16 of 116 women older than age 60 years (13.7%) were also found to have P/LP variants.

**Conclusions and Relevance:**

This large cohort study of women with breast cancer who underwent germline testing provided information about *BRCA1* and *BRCA2* in a population enriched for Hispanic women and reported the prevalence of P/LP variants when universal *BRCA1* and *BRCA2* testing is offered to women with breast cancer.

## Key Points

**Question:**

What is the prevalence of *BRCA1* and *BRCA2* pathogenic or likely (P/LP) variants in an unselected population of women with breast cancer?

**Findings:**

This cohort study enrolled 2401 women with breast cancer for germline testing. Hispanic women were 2.58 times as likely as non-Hispanic women to carry a P/LP germline mutation in *BRCA1* than *BRCA2* variants, and P/LP variants in *BRCA1* were identified in Hispanic women more often than in non-Hispanic women.

**Meaning:**

These findings suggest that universal BRCA1 and BRCA2 testing should be performed for all breast cancer patients, especially in women from minoritized racial and ethnic groups.

## Teaser

This cohort study examines the prevalence of pathogenic or likely pathogenic variants in *BRCA1* and *BRCA2* in women with breast cancer in a racially and ethnically diverse population.

## Introduction

Germline testing in women with breast cancer has been underused, especially in women from minoritized racial and ethnic groups (geographic location and ethnicity) in the determination of health disparities. Common barriers to germline testing include social determinants of health, fear of discrimination, physician understanding and bias, insurance concerns, cost, and time constraints for the medical team.^1,2,3^ Because the knowledge of *BRCA* status is critical to treatment planning of all stages of breast cancer, the argument for universal testing has been successfully made. The American Society of Clinical Oncology and Society of Surgical Oncology (ASCO) recently published guidelines for germline testing in patients with breast cancer.^4^ *BRCA1* and *BRCA2* testing should be offered to all patients with stage I to III or de novo stage IV or metastatic breast cancer who are aged 65 years or younger at diagnosis. Patients older than age 65 years should be offered *BRCA1* and *BRCA2* testing based on candidacy for poly (ADP-ribose) polymerase (PARP) inhibitors, personal or family history, and if they have triple negative disease. Patients of Ashkenazi Jewish ancestry or members of a population with an increased prevalence of founder mutations should also be offered *BRCA1 *and* BRCA2* testing.^4^

As part of the Implementing Next-generation Sequencing for Precision Intervention and Risk Evaluation study (INSPIRE) at City of Hope (COH), germline testing for 155 cancer genes is provided at no cost to the patient. All women with a diagnosis of breast cancer of any age are asked to participate in the INSPIRE study at Duarte and Upland facilities, which include a high number of women from underrepresented populations. This study examined the potential of a planned interim analysis to identify the prevalence of P/LP variants in a population of women with a breast cancer diagnosis including patients from underrepresented racial and ethnic minority group. We hypothesized that the results would provide insight into the prevalence of *BRCA1 *and* BRCA2* variants in the setting of universal testing.

## Methods

Patients with a breast cancer diagnosis were enrolled by the treating physician or a clinical research assistant to participate in the City of Hope’s INSPIRE institutional review board (IRB)–approved biorepository study from July 2020 to October 2023 at COH Duarte, California, and COH Upland, California, without consideration of age or limitations of consensus guidelines. This cohort study follows the Strengthening the Reporting of Observational Studies in Epidemiology (STROBE) reporting guideline. This study was conducted according to the principles of the Belmont Report: Ethical Principles and Guidelines for the Protection of Human Subjects or Research and the Declaration of Helsinki. The INSPIRE clinical trial was approved by the COH institutional review board. All patients completed written documentation of informed consent to participate, including the use of data and images for publication. To ensure that this component of the study was enriched for individuals from underrepresented populations, this unselected population of patients were self-reported to be African American or Black, American Indian or Alaska Native, Asian, Native Hawaiian or Other Pacific Islander, White, other (ie, patients who reported other), or unknown (ie, patients who declined to answer). Specific racial category included descent-associated population group descriptors (geographic location) for the determination of health disparities. Ethnicity was self-reported to be Hispanic or Latina, non-Hispanic, or unknown. Patients opted in for germline testing of 155 cancer genes without charge to the patient using blood, buccal smears, or saliva testing, and the results were reviewed by board-certified genetic counselors. Those individuals who were found to have a P/LP variant were contacted by a genetic counselor who reviewed the result and appropriate follow-up with a cancer geneticist. In this planned interim analysis, we selectively focused our report on the prevalence of *BRCA1* and *BRCA2* among women with breast cancer diagnosed between July 2020 and October 2023 in Duarte and Upland clinics at City of Hope.

### Molecular Analysis and Variant Classification

Genetic testing was performed at 1 of 2 College of American Pathologists-Clinical Laboratory Improvement Amendments certified commercial laboratories, either Fulgent Genetics (Temple City, CA) or Invitae Corp (San Francisco, CA). Specific analytic pipelines and variant classification from each of these correspond well, and any rare discordant classifications between laboratories were reviewed by a panel of genetic counselors and cancer geneticists for harmonization and consistent genetic counseling. Germline testing was performed for 155 cancer genes according to the test specifications from the laboratory. Variants are classified according to the 5-tiered categorization established by the American College of Medical Genetics: pathogenic, likely pathogenic, variant of uncertain significance, likely benign, or benign.^5^

### Statistical Analysis

Statistical analyses are performed in R programming language (version 4.4.1) for descriptive statistics and contingency table analyses to calculate odds ratios (ORs) and 95% CIs for comparisons by ethnicity and descent-associated descriptors (African American or Black, American Indian, Asian, Native Hawaiian or Other Pacific Islander, White, other, or unknown). Data were analyzed from October 2023 to December 2024. Statistical significance was set at *P* < .05.

## Results

A total of 2401 women with breast cancer at any stage 0 to IV (COH Duarte, California, and Upland, California) underwent germline testing, including 136 African American or Black women (5.7%), 365 Asian women (15.2%), 15 American Indian or Alaska Native women (0.5%), 8 Native Hawaiian or Other Pacific Islander women (0.3%), 1666 White women (69.4%), 86 women with other race (3.6%), and 125 women with unknown race (5.2%). Ethnicity was reported for 737 (30.7%) Hispanic or Latina women, 1545 (64.3%) non-Hispanic women, and 119 women with unknown ethnicity (5.0%). A total of 2401 women with breast cancer at any stage 0 to IV underwent germline testing, including 1934 from the main Duarte site (80.5%) and 467 from the Upland clinical network site (19.5%). The median (range) age was 54 (18-93) years. Patient characteristics are summarized in Table 1.

**Table 1.** Patient Characteristics

Characteristic	Patients, No. (%)	Patients, No. (%)			
		P/LP *BRCA1*	P/LP *BRCA2*	Either *BRCA1* or *BRCA2*	VUS or negative
Descent-associated descriptors					
African American or Black	136 (5.7)	7 (5.1)	4 (2.9)	11 (8.1)	125 (91.9)
American Indian or Alaska Native	15 (0.6)	0	1 (6.7)	1 (6.7)	14 (93.3)
Asian	365 (15.2)	5 (1.4)	11 (3.0)	16 (4.4)	349 (95.6)
Native Hawaiian or Other Pacific Islander	8 (0.3)	0	0	0	8 (100.0)
White	1666 (69.4)	36 (2.2)	44 (2.6)	80 (4.8)	1586 (95.2)
Other^a^	86 (3.6)	1 (1.2)	4 (4.7)^c^	4 (4.7)^c^	82 (95.3)
Unknown^b^	125 (5.2)	3 (2.4)	1 (0.8)	4 (3.2)	121 (96.8)
Ethnicity					
Hispanic	737 (30.7)	22 (3.0)	16 (2.2)^c^	37 (5.0)	700 (95.0)
Non-Hispanic	1545 (64.3)	25 (1.6)	47 (3.0)	72 (4.7)	1473 (95.3)
Unknown	119 (5.0)	5 (4.2)	2 (1.7)	7 (5.9)	112 (94.1)
Total	2401 (100.0)	52 (2.2)	65 (2.7)^c^	116 (4.8)^c^	2285 (95.2)

Abbreviations: P/LP, pathogenic or likely pathogenic variants; VUS, variants of uncertain significance.

^a^ Other includes women who checked “other” checkbox when provided the following list of options: African American or Black, American Indian or Alaska Native, Asian, Native Hawaiian or Other Pacific Islander, White, or Other.

^b^ Unknown includes women who declined to check a box.

^c^ One patient had both *BRCA1* and *BRCA2* variants.

BRCA1 and BRCA2 pathogenic or likely pathogenic (P/LP) variants are reported using descent-associated descriptors and ethnicity. P/LP variants in *BRCA1* or *BRCA2* were identified in 11 of 136 African American or Black women (8.1%), 1 of 15 (6.7%) American Indian and Alaska Native, 16 of 365 Asian women (4.4%), 4 of 86 women with other race (4.7%), 0 of 8 Native Hawaiian or Other Pacific Islander women, 80 of 1666 White women (4.8%), and 4 women with unknown race (3.2%). Table 2 reported the prevalence of P/LP variants in *BRCA1* that were more likely to be identified in 22 of 737 Hispanic women (3.0%) compared with 25 of 1545 non-Hispanic women (1.6%) (OR, 1.87; 95% CI, 1.04-3.34; *P* = .02). Table 3 shows Hispanic women with breast cancer were 2.58 times as likely as non-Hispanic women to carry a P/LP germline *BRCA1* than *BRCA2* mutation (OR, 2.58; 95% CI, 1.16-5.88; *P* = .02). The prevalence of *BRCA2* variants were comparable for Hispanic and non-Hispanic women. Six of the deleterious variants found in this cohort (BRCA1IVS5 + 1G>A, 186delAG, 1135insA, A17008E, BRCA2 3492insT, and 9254del5) have been reported in the literature in families of Hispanic ancestry.^6,7^

**Table 2.** Prevalence of Individual P/LP Variants by Ethnicity^a^

Characteristic	*BRCA1*, No. (%)		OR (95% CI)	*P* value	*BRCA2*, No. (%)		OR (95% CI)	*P* value
	P/LP	VUS/Negative			P/LP	VUS or negative		
Hispanic	22 (3.0)	715 (97.0)	1.87 (1.04-3.34)	.03	16 (2.2)	721 (97.8)	0.71 (0.39-1.23)	.24
Non-Hispanic	25 (1.6)	1520 (98.4)	1 [Reference]		47 (3.0)	1498 (97.0)	1 [Reference]	

Abbreviations: OR, odds ratio; P/LP, pathogenic or likely pathogenic variants; VUS, variant of uncertain significance.

^a^ Hispanic women with breast cancer are 1.87 times as likely as non-Hispanic women to carry P/LP *BRCA1* variants. The prevalence of *BRCA2* was comparable for Hispanic and non-Hispanic women.

**Table 3.** Prevalence of P/LP Variants by Ethnicity^a^

Characteristic	No. (%) with P/LP variant		OR (95% CI)	*P* value
	*BRCA1*, No. (%)	*BRCA2*, No. (%)		
P/LP mutation	P/LP mutation
Hispanic	22 (57.9)	16 (42.1)	2.58 (1.16-5.88)	.02
Non-Hispanic	25 (34.7)	47 (65.3)	1 [Reference]	

Abbreviations: OR, odds ratio; P/LP, pathogenic or likely pathogenic variants.

^a^ Hispanic women with breast cancer are 2.58 times as likely as non-Hispanic women to carry a P/LP germline *BRCA1* mutation than *BRCA2* mutation.

Table 4 reports the prevalence of *BRCA1* and *BRCA2* variants of 8.1% among African American and Black women with breast cancer is high and clinically important to recognize, especially in the setting of historic obstacles to achieving appropriate rates of testing in this group. Here, African American or Black women with breast cancer were approximately 1.74 times as likely as White women (OR, 1.74; 95% CI, 0.91-3.36) to carry a P/LP germline mutation in either *BRCA1* or *BRCA2*. However, this increase in risk does not reach a nominal threshold of statistical significance, and 95% CIs include the null hypothesis of no association (OR = 1.00).

**Table 4.** Prevalence of P/LP Variants in *BRCA1* or *BRCA2* Among African American or Black and White Women With Breast Cancer^a^

Characteristic	No. (%) with *BRCA1* or *BRCA2*		OR (95% CI)	*P* value
	P/LP	VUS or negative		
African American or Black	11 (8.1)	123 (91.9)	1.74 (0.91-3.36)	.09
White	80 (4.8)	1586 (95.2)	1 [Reference]	

Abbreviations: OR, odds ratio; P/LP, pathogenic or likely pathogenic mutation; VUS, variants of uncertain significance.

^a^ African American or Black women with breast cancer are 1.74 times as likely as White women to carry a P/LP germline mutation in either *BRCA1* or *BRCA2*, although this increase in risk does not reach a nominal threshold of statistical significance and 95% CIs include the null hypothesis of no association (OR = 1.00).

Of the 47 women with known ethnicity carrying a P/LP *BRCA1* mutation, 22 of 47 were Hispanic (46.8%). Of the 63 women with known ethnicity carrying a P/LP *BRCA2* mutation, 16 of 63 were Hispanic (25.4%) (1 patient had both *BRCA1* and *BRCA2* variants). The age of patients by carrier status and ethnicity is shown in the Figure. A total of 16 women aged 60 years or older (13.7%) were found to have a P/LP mutation in either *BRCA1* or *BRCA2*. Specific pathogenic *BRCA1* and *BRCA2* variants are shown in the Figure by ethnicity.

**Figure.**
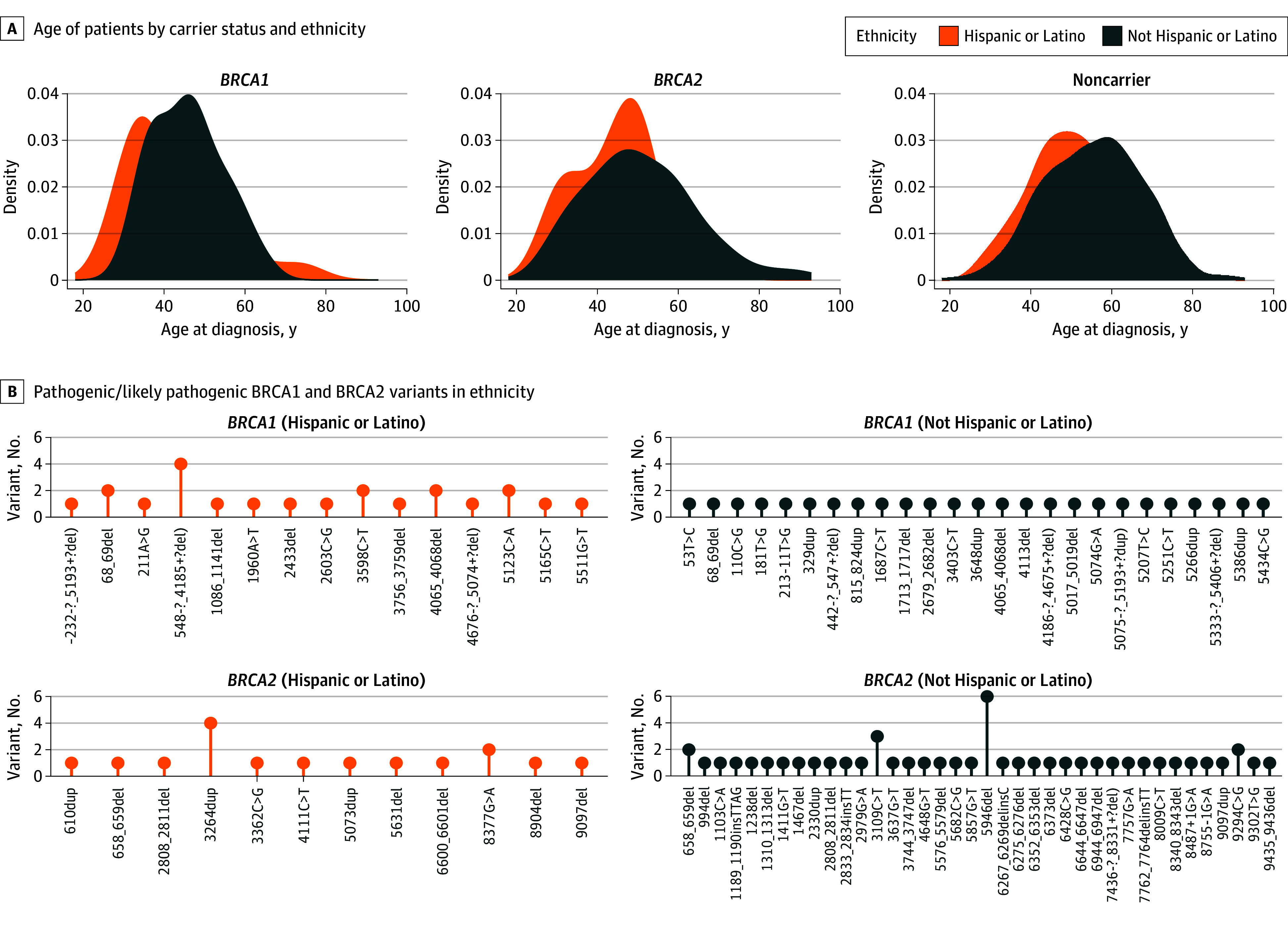
Patient Characteristics (A) Age of patients by carrier status and ethnicity. A total of 16 (13.7%) women ≥60 years of age were found to have a pathogenic or likely pathogenic mutation in either *BRCA1* or *BRCA2*. (B) Pathogenic or likely pathogenic *BRCA1* and *BRCA2* variants by ethnicity.

## Discussion

For years germline testing has been restricted by guidelines developed by a consensus of leaders in the field. This was appropriate because of several barriers, including the high cost of testing, access to appropriate specialists, and the potential legal and psychological stigma attached to the finding of a hereditary disposition. Over time, these barriers have been broken down. The cost of germline testing has become less cost prohibitive, and direct to consumer testing is readily available and has heightened the awareness and appreciation for this information.^8^ Knowledge of *BRCA1* and *BRCA2* status now informs treatment decisions at all stages of disease from the initial surgical planning to adjuvant therapy, as well as treatment of advanced disease. Because of this, physicians are increasingly comfortable ordering these tests. It is appropriate that the recently released American Society of Clinical Oncology/Surgical Society of Oncology genetic testing guidelines endorse germline testing for *BRCA1* and *BRCA2* in all newly diagnosed women with breast cancer who are younger than age of 65 years. The guidelines also specifically include testing women older than age 65 years based on family history, Ashkenazi Jewish heritage, diagnosis of triple negative disease, and candidacy for PARP inhibitors.^4^

This cohort study analyzed data from a large cohort of women with a diagnosis of breast cancer regardless of age, and we identified pathogenic or likely pathogenic variants in *BRCA1* and *BRCA2* in 4.4% of Asian patients, 8.1% of African American or Black patients, and 4.8% of White patients. The ethnic breakdown of our cohort represented our catchment area with 31% of the women self-identifying as Hispanic. The prevalence of variants among the 747 women who self-identified as Hispanic was 5.0% and was comparable to the 4.7% identified in non-Hispanic women. Hispanic women were 1.87 times more likely to carry a P/LP *BRCA1* variant than non-Hispanic women while the prevalence of *BRCA2* variants was similar. Hispanic women with breast cancer were 2.58 times as likely as non-Hispanic women to carry a P/LP germline *BRCA1* mutation than *BRCA2* mutation. Six of the deleterious mutations found in this cohort (BRCA1IVS5 + 1G>A, 186delAG, 1135insA, A17008E, BRCA2 3492insT, 9254del5) have been reported in the literature in families of Hispanic ancestry.^6,7^ This is partially attributable to known Mexican founder mutations in our unselected Southern California population. All women with a breast cancer diagnosis were included in this study regardless of age. Of 116 women who were aged 60 years and older, 16 (13.7%) were found to have a P/LP mutation of *BRCA1* and *BRCA2*. The prevalence of *BRCA1* and *BRCA2* P/LP of 4.8% reported in our series of 2401 patients is comparable to what has been reported in other reports of universal genetic testing in women with breast cancer.^9,10^

African American or Black women with breast cancer have historically been less likely to undergo *BRCA1 *and* BRCA2* testing than White women, with a population-based study finding that African American or Black women were 60% less likely to undergo testing than White women (OR, 0.40; 95% CI, 0.34-0.48).^11,12^ This study found that the prevalence of *BRCA1* and *BRCA2* pathogenic or likely pathogenic variants may be even more common in African American or Black women with breast cancer than White, whereas African American or Black patients had a higher prevalence of pathogenic variants in *BRCA2* (1.80% vs 1.24%; *P* = .005).^13,14,15^ In this study, we reduced these barriers to testing in a research setting by subsidizing no cost testing for all patients with breast cancer, regardless of age, race, ethnicity, guideline-based criteria, insurance coverage or social determinants of health. Our study provided additional evidence and supports the conclusion that guidelines should be updated and revised to assure equitable access to genetic testing for all women with breast cancer.

### Limitations

The argument for universal germline testing in women with breast cancer has been effectively made with the recent publication of the ASCO/SSO guidelines. Our study is limited by lack of knowledge as to how the status of P/LP *BRCA1* and *BRCA2* variants impacted treatment of individual patients with breast cancer or how many patients took advantage of cascade testing. Another limitation is selection bias due to motivation of women to consent to germline testing and other potential confounders.

## Conclusion

This large cohort study provided information about *BRCA1* and *BRCA2* in a population with a substantial number of Hispanic women and a glimpse of findings when universal *BRCA1* and *BRCA2* testing is offered. These findings suggest that universal BRCA1 and BRCA2 testing should be performed for all breast cancer patients, especially in women from minoritized racial and ethnic groups.

## Notes

Supplement.
Data Sharing Statement
