# Supplementary material for: Prevalence of BRCA1 and BRCA2 Variants in an Unselected Population of Women With Breast Cancer
Source: JAMA Netw Open. 2025 Sep 15;8(9):e2531577. doi: 10.1001/jamanetworkopen.2025.31577 (PMC12439053; doi:10.1001/jamanetworkopen.2025.31577)
Supplement: Supplement. — Data Sharing Statement [file jamanetwopen-e2531577-s001.pdf]

Data Sharing Statement

Mortimer. Prevalence of BRCA1 and BRCA2 Variants in an Unselected Population of Women With Breast Cancer. *JAMA Netw Open*. Published September 15, 2025. doi:10.1001/jamanetworkopen.2025.31577

Data
Data available: No

Additional Information
Explanation for why data not available: Data privacy and confidentiality preclude data sharing due to human participants and sensitive information that may compromise ethical standards or legal requirements.
